# Analysis of Incidence and Related Factors of Hemorrhagic Fever with Renal Syndrome in Hebei Province, China

**DOI:** 10.1371/journal.pone.0101348

**Published:** 2014-07-07

**Authors:** Qi Li, WenNa Zhao, YaMei Wei, Xu Han, ZhanYing Han, YanBo Zhang, ShunXiang Qi, YongGang Xu

**Affiliations:** Institute for Viral Disease Prevention and Control, Hebei Province Centre for Disease Prevention and Control, Shijiazhuang, Hebei Province, China; University of Texas Medical Branch, United States of America

## Abstract

**Background:**

Hemorrhagic fever with renal syndrome (HFRS) is an important infectious disease in Hebei province, China. The average annual incidence is 3.10/100000. Although the incidence of HFRS is stable with a general decline in trend since 2004, an increase in the trend was observed in 2011. Few studies have been conducted to investigate the underlying risk factors for this disease.

**Methods:**

The epidemiological data of HFRS and the population data, meteorology, and remote sensing aspects of cities in Hebei province from 1999 to 2011 were collected. The epidemiological data included the population density in the region, seasonal data and rat density and rat's virus carriage rate. The data were analyzed by descriptive study, correlation analysis and multivariate linear analysis.

**Results:**

There were 26837 cases of HFRS was reported from 1999 to 2011. The infection occurred mainly in males, aged 20∼49 years, who were farmers. *Rattus norvegicus* was the main host animal. The incidence of HFRS was related to NDVI value, rat density and rat's virus carriage rate by multiple linear regression (*F* = 25.936, *P*<0.01). The pseudo- *R* ^2^value for the model was 0.644.

**Conclusion:**

The incidence of HFRS was related to NDVI value, rat density and rat's virus carriage rate. Control of these factors should be used to prevent HFRS in Hebei province.

## Introduction

Hemorrhagic fever with renal syndrome (HFRS) is caused by different species of Hantavirus which causes fever, bleeding and renal dysfunction. HFRS is characterized by high mortality and is a serious public health problem. The annual number of cases reported in China is 40000-60000, which accounts for 90% of the total reported cases of HFRS throughout the world [1]. HFRS has been reported in 32 provinces in China [2].

The incidence and prevalence of HFRS can be affected by environmental, occupational, population and reservoir factors [3]. A number of studies have examined the correlation between HFRS and meteorological factors [4]–[6]. Meteorological factors, including temperature, rainfall and relative humidity, affect the replication rate of the virus. It can also effect the disease reservoir, rodents, and contacts between human and rodent populations [7]. The Normalized Differential Vegetation Index (NDVI) reflects the growth of vegetation [8]. There is a significant correlation between the incidence of HFRS and NDVI in monitoring areas of China [9].

In Hebei province, the first case of HFRS was reproted in Tangshan, in 1981. Since then, HFRS cases have been reported every year. The average annual incidence is 3.10/100000 and the cases have spread to 152 counties (urban). This accounts for 86.36% of all counties (urban) in Hebei province. Rat control and public education have been used to help control HFRS in Hebei province. The incidence has fluctuated since 1981, with periods of stabilization and decline since 2004, followed by an increase in 2011. There are few studies evaluating risk factors for disease.

We analyzed the epidemiologic characteristics of HFRS and explored the related factors, including meteorological factors, NDVI, rat density and rat's virus carriage rate.

## Methods

### Data collection and management

**Figure 1:**
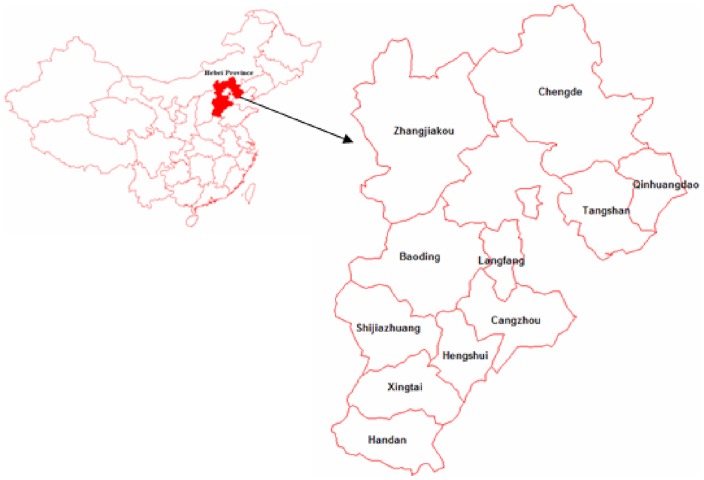
The location of Hebei province.

The epidemiologic data of cases with HFRS and the data regarding population, meteorology, and remote sensing in cities of Hebei province between 1999 and 2011 were collected. The epidemiologic data included the incidence of HFRS, rat density and rat's virus carriage rate. Fig. 1 shows the location of Hebei province in China.

All the epidemiologic data, including incidence and host animal information, were provided by the Hebei Centre for Disease Control and Prevention. Local meteorological data, including yearly precipitation, average temperature, number of days of sunshine and remote sensing data for the study period were obtained from the Institute of Meteorological Sciences of Hebei province.

The NDVI was derived using Advanced Very High Resolution Radiometer (AVHRR) images covering latitude 36° to 43° north and longitude 113° to 120° east. Annual NDVI from 1999 to 2011 were calculated using remote sensor monitoring systems of meteorological satellite V1.0, which was developed by the Institute of Meteorological Sciences of Hebei province. The annual NDVI of cities in Hebei province was calculated using an electronic map scale of 1:250000.

### Statistical analysis

SPSS13.0 software was used to explore associations between HFRS incidence and relevant factors including rat density, rat's virus carriage rate, annual NDVI, yearly precipitation, yearly average temperature, and number of sunny days per year. A multivariate linear analysis was performed to build a model using the variables rat density(X_1_) and rat's virus carriage rate (X_2_). Other variables were tested to see if they would improve the model fit. Adding the variable annual NDVI(X_3_) did lead to a more accurate model.

## Results

**Figure 2:**
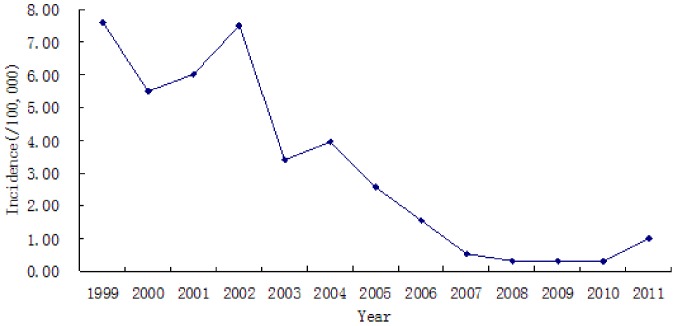
The incidence of HFRS in Hebei province from 1999 to 2011.

26837 HFRS cases were reported between1999 and 2011. The average annual incidence was 3.10/100000. The epidemic situation presented a decline tendency obviously. The incidence has presented low levels since 2004, and maintained less than 1/100000 in recent years. An increasing trend was observed in 2011 (Fig.2)

HFRS cases were reported in all age groups, the largest number was seen in individuals aged 20–49 years, accounting for 65.53% of the total. The male vs female incidence ratio, 2.49:1, remained relatively constant over the study period. Most affected individuals were employed as farmers, students, workers and cadre staffs. Mainly farmers accounted for 74.64% of affected individuals.

**Figure 3:**
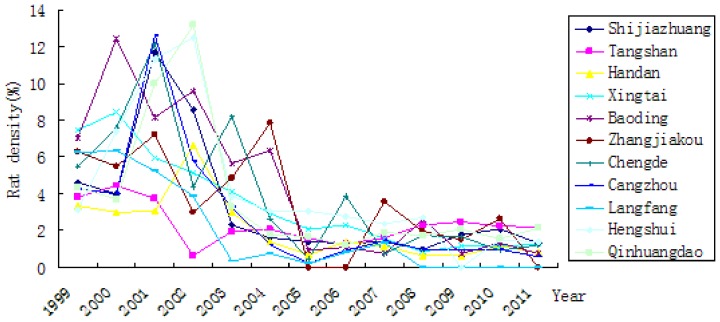
Rat density in cities of Hebei province from 1999 to 2011.

**Figure 4:**
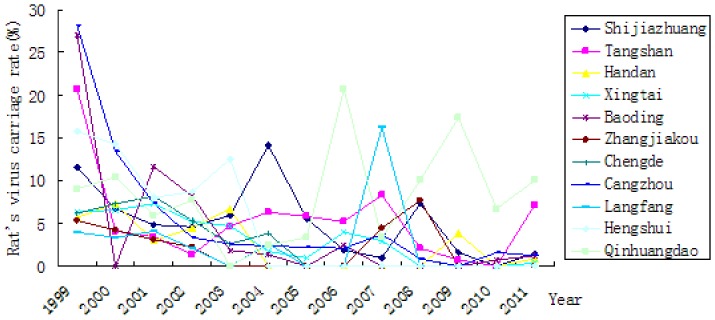
Rat's virus carriage rate in cities of Hebei province from 1999 to 2011.

The rat density decreased over the time of the study (Table 1, Fig. 3). The rat's virus carriage rate varied over time (Table 1, Fig. 4). Through classifying and identifying the composition of rodent in Hebei province, *Rattus norvegicus* was the main host animal identified in both residential and field areas. There was significant difference in the rat density in residential area and field areas (χ2 = 218.770, *P*<0.01).

**Table 1:** Rat density and rat's virus carriage rate in cities of Hebei province from 1999 to 2011.

	Rat density		Rat's virus carriage rate	
Year	Residential area (%)	Field area (%)	Residential area (%)	Field area (%)
1999	6.02	9.08	3.69	5.43
2000	5.4	8.53	3.14	8.79
2001	4.86	10.08	2.49	6.12
2002	3.99	7.71	1.92	5.2
2003	3.37	2.23	3.89	4.27
2004	3.38	1.89	3.39	3.06
2005	1.48	0.99	4.62	0.97
2006	2.08	1.19	5.24	1.18
2007	2.18	1.36	2.82	1.44
2008	2.62	1.24	3.63	1
2009	2.02	0.52	3.02	0.89
2010	2.08	1.27	1.96	0.84
2011	2.01	0.7	7.46	0

According to the matrix scatter, we knew that natural logarithm of incidence and two variables of rat density and rat's virus carriage rate was basically in linear distribution. And most of the standardized residuals obeyed normal distribution depended on normal probablity plot of standardized residual plots. Through making residuals plot, it showed that standardized residual did not vary with the change of values of variable.

The rat density and the rat's virus carriage rate were used to as variables in a multiple linear regression analysis. All variables with statistical significance were entered into the regression model (F = 78.252, P<0.01). The pseudo-R^2^value for the first model was 0.608 (Table 2, Table 3). The data in this study met the requirements for the application of multivariate linear regression model above.

**Table 2:** Correlation of the natural logarithm of incidence of HFRS, with meteorological factors, NDVI, rat density and rat's virus carriage rate in cities of Hebei Province.

	Correlation coefficient	Natural logarithm of incidence	Rat density	Rat's virus carriage rate	Precipitation	Mean temperature	Days of sunshine	Average NDVI values
Natural logarithm of incidence	r	1.000	0.558*	0.663*	−0.051	0.185*	0.007	−0.463*
	p	.	0.000	0.000	0.314	0.038	0.475	0.000
Rat density	r		1.000	0.234*	−0.199*	0.090	0.007	−0.376*
	p		.	0.012	0.028	0.197	0.475	0.000
Rat's virus carriage rate	r			1.000	0.055	0.107	0.126	−0.261*
	p			.	0.301	0.153	0.114	0.006
Precipitation	r				1.000	0.182*	−0.143	0.205*
	p				.	0.040	0.086	0.024
Mean temperature	r					1.000	−0.641*	−0.247*
	p					.	0.000	0.008
Days of sunshine	r						1.000	0.141
	p						.	0.089
Average NDVI values	r							1.000
	p							.

Note:*represents *P*<0.05.

**Table 3:** Linear regression model of natural logarithm of annual incidence of HFRS and related factors in cities of Hebei province from 1999 to 2011.

Regression equation	R	R^2^	F value	P value	Durbin-Watson
y = −1.501+0.229X_1_+0.146X_2_	0.780	0.608	78.252	0.000	1.668
y = −0.668+0.188X_1_+0.127X_2_−0.054X_3_	0.803	0.644	25.936	0.000	1.839

Adding the variable mean temperature did not improve the fit (t = 0.488,*P*>0.05) (Table 2). Rat density, rat's virus carriage rate and NDVI value contributed to form the most accurate model (*F* = 25.936, *P*<0.01). The pseudo- *R* ^2^value for the second model was 0.644 (Table 3).

**Figure 5:**
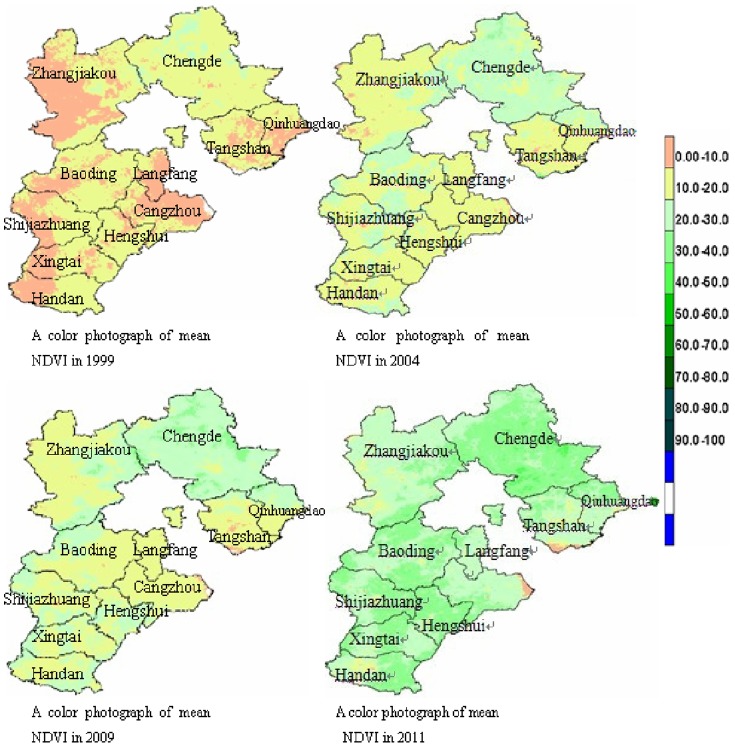
The mean NDVI of cities in Hebei province in 1999, 2004, 2009 and 2011.

The yearly incidence of HFRS correlated positively with rat density and rat's virus carriage rate. The average NDVI value increased year by year (Fig. 5). javascript:;There was a negative correlation between average incidence and the average yearly NDVI value in cities of Hebei province (*r* = −0.463, *P*<0.05). There was no significant correlation with mean temperature, inches of precipitation or number of days of sunshine.

## Discussion

Previous studies have shown a relationship between HFRS and social and natural factors including rat density, climate, human population, and life condition [10]. This study also evaluated factors associated with pathogen transmission, including rat infection rate and population setting. The data were summarized and analyzed using a descriptive study and correlation analysis.

NDVI value, average temperature, rat density and rat's virus carriage rate were evaluated for their relationship with HFRS infection. All variables entered into the regression model, except for average temperature, showed a correlation with HFRS infection (*R* ^2^ = 0.644). Variables used in the regression model contributed to 64.4% of the variation of HFRS occurrence in cities of Hebei province. The current epidemiologic surveillance date regarding HFRS in Hebei province was not complete, so the model could not be used to forecast epidemics.

Remote sensing technology has been used to study the epidemiology of vector-borne infectious disease such as dysentery, and schistosomiasis [11]–[12]. When the NDVI was poor and vegetation coverage was low, rats could not survive. A large number of *Rattus norvegicus* would then migrate from farm areas to urban areas, leading to an increase in the rate of HFRS.

Previous investigations have shown that the prevalence of HFRS was related to climate changes [13]. The amount of rain and temperature can affect rat breeding. The relationship between days of sunshine and the incidence of HFRS is not clear. However, we did not find a relationship between precipitation, temperature and number of days of sunshine, and prevalence of HFRS.

Hebei province is located on the plains of northern China, host to an indigenous population of *Ruttus norvegicus*. We found a correlation between the rat density and the yearly incidence of HFRS. Previous reports suggest that controlling the rat density to less than 1% can control the spread of HFRS [14]–[15]. Rat proofing and deratization are standard methods used to control HFRS. Increased public awareness regarding disease prevention, improved living accommodations, and enhanced anti infection ability are also needed to decrease the infection rate of HFRS.

The occurrence and prevalence of HFRS is not only closely related to natural factors, but also to social factors and people's behavior. The ralationship of these factors should be considered when evaluating risk factors and making prediction [16]. Thus, the study showed that the incidence of HFRS was related to NDVI value, rat density and rat's virus carriage rate. It have important value to the epidemics' control.
